# Encouraging the scale-up of proven interventions: Infrastructure development for the “Evidence-to-Implementation” award

**DOI:** 10.1017/cts.2021.828

**Published:** 2021-07-26

**Authors:** Andrew Quanbeck, Roberta A. Johnson, Mondira Saha-Muldowney, Felice Resnik, Sheena Hirschfield, Rachael R. Meline, Jane E. Mahoney

**Affiliations:** 1 D & I (Dissemination and Implementation) Launchpad, Institute for Clinical and Translational Research, School of Medicine and Public Health, University of Wisconsin – Madison, Madison, WI, USA; 2 Department of Family Medicine and Community Health, School of Medicine and Public Health, University of Wisconsin – Madison, Madison, WI, USA; 3 Division of Geriatrics and Gerontology, Department of Medicine, School of Medicine and Public Health, University of Wisconsin – Madison, Madison, WI, USA

**Keywords:** Implementation, non-patentable interventions, entrepreneurism, commercialization, late-stage translation

## Abstract

**Background/Objective:**

Although most research universities offer investigators help in obtaining patents for inventions, investigators generally have few resources for scaling up non-patentable innovations, such as health behavior change interventions. In 2017, the dissemination and implementation (D & I) team at the University of Wisconsin’s Clinical and Translational Science Award (CTSA) created the Evidence-to-Implementation (E2I) award to encourage the scale-up of proven, non-patentable health interventions. The award was intended to give investigators financial support and business expertise to prepare evidence-based interventions for scale-up.

**Methods:**

The D & I team adapted a set of criteria named Critical Factors Assessment, which has proven effective in predicting the success of entrepreneurial ventures outside the health care environment, to use as review criteria for the program. In March 2018 and February 2020, multidisciplinary panels assessed proposals using a review process loosely based on the one used by the NIH for grant proposals, replacing the traditional NIH scoring criteria with the eight predictive factors included in Critical Factors Assessment.

**Results:**

two applications in 2018 and three applications in 2020 earned awards. Funding has ended for the first two awardees, and both innovations have advanced successfully.

**Conclusion:**

Late-stage translation, though often overlooked by the academic community, is essential to maximizing the overall impact of the science generated by CTSAs. The Evidence-to-implementation award provides a working model for supporting late-stage translation within a CTSA environment.

## Introduction

The University of Wisconsin – Madison (UW) is one of the nation’s 61 NIH-funded Clinical and Translational Science Award (CTSA) grantees. The name of the grantee at UW is the Institute for Clinical and Translational Research (ICTR). The goal of ICTR is to create an environment at UW that facilitates the conduct of research that improves human health. Some research conducted at UW results in patentable products, such as drugs and devices, and the UW – like almost all research universities – has robust technology transfer infrastructure in place to help researchers apply for patents on their inventions and create new businesses (e.g., the Wisconsin Alumni Research Foundation and Discovery to Product). But UW – again like most research universities – has less infrastructure to support scaling up non-patentable interventions (e.g., novel health care models, behavioral or other health interventions, smartphone applications, etc.) that have been shown to improve public health, representing a potential evidence-to-practice gap to be closed.

The Evidence-to-Implementation (E2I) award at UW was designed to speed the uptake of non-patentable evidence-based practices by bridging the gap between academic research and financially self-sustaining success in the marketplace. The purpose of this article is to describe the elements, processes, and tools used in the E2I award program that are intended to support late-stage translation within a CTSA environment, to share early results on the feasibility and acceptability of the program gathered from participant feedback to guide decisions for adoption at other CTSA sites, and to outline plans for future evaluation of the model. One important obstacle to the wide use of research-based innovations is that investigators in medicine, health services, engineering, nursing, pharmacy, and related fields often lack background in the tools of business, such as assessing the market for an innovation, developing a business plan, and creating promotional materials. Further, most investigators have limited access to purveyors or intermediary organizations that might help commercialize the interventions they’ve developed. [1] A *purveyor* is “an individual or group of individuals representing a program or practice who actively work to implement that practice or program with fidelity and good effect.” [2] Intermediary organizations have been characterized as “… developing, implementing, and supporting multiple best practice programs or services, as well as building the capacity within an agency or system in order to implement and sustain such programs.” [3,4] In this manuscript, we use *purveyor* to refer to both types of organizations. The E2I award is designed to address the unmet needs of researchers aiming to scale up their evidence-based innovations.

## Methods

### Needs Addressed by the E2I Program

The E2I award evolved from a previous award at UW called the Dissemination Supplement Award. From 2014 to 2016, ICTR funded the Dissemination Supplement Award to help researchers create materials to spread the use of their innovations. Nine awards were made between 2014 and 2016. The award helped researchers create toolkits that would enable adopters to implement the innovations; the toolkits are available at the Health Innovation Program exchange website (https://www.hipxchange.org). The Dissemination Supplement program did not provide support to help launch innovations that require more than a toolkit to reach wide use, and a stakeholder-driven evaluation revealed other types of support investigators needed as well. Table 1 shows those needs and how the E2I award was configured to meet them.

**Table 1.** Unmet needs in Dissemination Supplement award and resulting features of the Evidence-to-Implementation (E2I) award

Unmet need	Resulting feature(s) in E2I award
*Investigator’s team:*	
A mechanism that allows investigators interested in scale-up to nominate themselves for funding support	The E2I is a competitive award so that interested investigators can apply for funding (rather than being sought out).
A way to ensure the investigator’s team has the protected time and capacity to scale up	Investigators are required to commit to seeing the project through to scale-up through formal letter of commitment. Awardees receive $75,000 in direct costs and 18 months of in-kind support.
A way to compensate the investigator and team for their implementation efforts	
*Intervention:*	
A method that enables investigators to assess the demand for their innovations	Dissemination and implementation (D & I) Launchpad staff members provide an initial assessment of market demand before the award is made. Ongoing assessment is supported post-award.
*Process:*	
A method to identify business, implementation, and marketing needs for scale-up	D & I Launchpad staff members with business, marketing, and implementation science expertise offer a consultation service to help investigators prepare their applications. The team also gives awardees extensive implementation support for 18 months post-award.
An understanding between the award program and the investigator that makes goals and deliverables clear	As part of the application, expectations of awardees and D & I Launchpad staff members are specified in a mutually agreed-upon work plan with milestones.
Support at the various stages of preparation for scale-up	Awardees receive 18 months of in-kind support from D & I Launchpad staff.

The E2I award is administered by a program within ICTR called the D & I (Dissemination and Implementation) Launchpad. The D & I Launchpad is designed to increase the use of evidence-based practices from research by offering education and training, consultation, help with packaging and communication, implementation support, and other services to investigators campus-wide. Launchpad staff members have worked in business, health care, and academic environments and have experience in marketing, finance, sales, product development, and social media as well as in implementation science.

### Use of a Tool to Predict the Likelihood of Scale-up Success

In addition to addressing the needs shown in Table 1, the E2I program attempts to answer a question that arises whenever scarce resources must be allocated: Which ventures are likely to fail and which succeed? For the E2I award, the D & I Launchpad team adapted a scoring method from an evidence-based predictive tool that was designed to determine the probability of the long-term success of entrepreneurial ventures. The tool, called the Critical Factor Assessment, uses a 37-factor predictive model that was developed by the Canadian Innovation Centre and shown to have strong predictive value in forecasting commercial success or failure by Astebro and Elhedhli. [5] We adapted a reduced, eight-factor version of the Critical Factor Assessment named the “CFA Snapshot” that is publicly available on the Canadian Innovation Centre website (www.innovationcentre.ca). It includes self-assessment criteria that prospective entrepeneurs can use to gauge the commercial viability of their proposed ventures. Table 2 shows the eight criteria included in the CFA Snapshot and the corresponding factor (adapted from the CFA Snapshot factors) used in the E2I application and review process.

**Table 2.** Critical factor assessment snapshot elements and Evidence-to-Implementation (E2I) scoring criteria

Critical Factor Assessment Snapshot element	E2I scoring criteria
1. Feature and benefits Does your proposed product or service offer performance advantages compared to currently deployed solutions?	1. Features and benefits - Does the innovation offer performance advantages compared with current solutions? - Would the innovation create value for implementing organizations? - Is the innovation evidence-based or does it have scientific merit?
2. Readiness How far away are you from being able to deliver completed products or services to your first revenue customer?	2. Market readiness - Has the innovation gone through different development stages? - What steps have been taken to prepare for broad dissemination?
3. Barrier to entry What is unique or patentable about your product that represents a barrier to entry to potential competitors?	3. Competition and barrier to entry - What will create an ongoing competitive advantage for the innovation? - What is unique about it?
4. Adoption Can you demonstrate that customers in your target market will purchase your product or service when it is available?	4. Demand/adoption/target customer - Has an adopter or first customer been identified? - Have potential adopters or customers been involved in development? - Is there evidence of demand?
5. Supply chain Can you provide confirmation that there are no success barriers either about your supply chain or distribution channel?	5. Sustainability – purveyors and partners - Is a purveyor willing to represent the product and take it to new customers? - Are partners willing to collaborate with the investigator?
6. Market size Is the overall size of the market and your likely market share sufficient to generate the envisaged revenues? Further, is the overall market forecast large enough to be interesting?	6. Potential for impact - Will the innovation impact the health of individuals, improve care processes, and/or increase safety or efficiency? - Is the innovation likely to benefit a large audience or a subset of the population?
7. Entrepreneur experience Do you or members of your team have any direct or relevant (entrepreneurial, industry and business) experience that can be directly applied to the challenges facing this business?	7. Investigator and team Do the investigator and team have the expertise, resources, ability, and motivation to navigate the challenges to optimize uptake of the innovation, as evidenced by previous experience?
8. Financial expectations Do your financial projections present a persuasive argument that your company can achieve cash flow neutrality, based on your own investment, money you can borrow, and money you can raise from external investors?	8. Sustainability – finances - Is the innovation feasible? Is it acceptable to target audiences? - Does it have the potential to sustainably produce revenue for the purveyor?

During the proposal-writing process, the D & I Launchpad team helps investigators preliminarily answer the questions shown in the right column of Table 2. For example, team members offer technical assistance and make calls to potential health care adopters on behalf of investigators to inquire about implementation feasibility. The goal of the work is to provide feedback that might improve investigators’ prospects of scaling up their innovations, whether or not they succeed in getting an E2I award.

### Resources provided to grantees

The D&I Launchpad and the E2I award received dual funding from the UW’s CTSI and matching funds from the Wisconsin Partnership Program, an entity formed in 2004 through the Blue Cross/Blue Shield conversion that has a mandate to support public health in the state of Wisconsin. The D&I Launchpad is staffed by a program manager, a marketing specialist, an implementation scientist, and an admininstrative support person whose salaries are supported by both the CTSA and the Wisconsin Partnership Program. The E2I award funds $75,000 in direct costs, part of which is meant to enable the principal investigator(s) to take part in the iterative process of preparing an innovation for scale-up. Funding can also be used for website development, beta testing of the implementation package, and other scale-up activities. In addition to direct costs, the award gives in-kind support by D & I Launchpad staff for up to 18 months. In-kind support includes services related to preparing a business plan, including assessing market sector(s) and conducting market research; identifying possible purveyors; refining the value proposition for purveyors, adopters, and end users; writing a pricing plan; developing branding, marketing, and training materials; and facilitating compliance with intellectual property law. The goal of the work is to disseminate the innovation either by UW or a third-party purveyor approved by UW.

The request for applications (RFA) for the E2I award describes each step of the application process. Researchers who have developed a successful innovation that meets a specific need in health care or the community are encouraged to apply. The 2021 RFA is shown in Supplementary File 1; the RFA is also available at www.hipxchange.org/Evidence-to-Implementation-Award. The RFA is announced to ICTR’s partners through various media in the UW schools of medicine and public health, nursing, pharmacy, and veterinary medicine, the College of Engineering, and at the Marshfield Clinic (a health system in rural northern Wisconsin affiliated with ICTR). Past recipients of ICTR’s translational research awards also receive emails about the RFA. Applicants must commit to collaborating with the D & I Launchpad team to develop the scale-up package, which consists of materials necessary for moving the innovation into the market, provide impact metrics for at least three years after completing the award, and work with stakeholders, partners, purveyors, and others to ensure the sustainability of the innovation. Throughout the application process and in subsequent work with award recipients, D & I Launchpad staff members work closely with investigators to ensure they remain compliant with all UW and state of Wisconsin regulations pertaining to conflicts of interest.

## The Application Process

### Step 1: Workshop

Applicants are required to attend a 60-minute workshop that covers the application process and timeline, the required components of the pre-proposal and application, and information about permitted and prohibited uses of E2I funds.

### Step 2: Pre-Proposal

Pre-proposals are due about a month after the workshop takes place. The purpose of the pre-proposal is to help both investigators and the D & I Launchpad team assess whether the innovation is a good fit for the award. Applicants prepare a three-page pre-proposal that addresses the eight criteria adapted from the Critical Factors Assessment and ultimately used by the review committee to rate applications. Applicants are asked to identify the features and benefits of their innovation, the problem or gap the innovation addresses, the value the innovation offers over already-available solutions, the scientific merit of the innovation, and the target audience. Applicants are not expected to have fully developed responses to all eight factors at this stage. One week after the pre-proposal submission deadline, researchers with innovations that align with the goals of the program are invited to move to Step 3.

### Step 3: Proposal Development

The proposal due is 6 to 8 weeks after applicants are invited to move to Step 3. To develop full proposals, applicants are required to meet once with the D & I Launchpad team and may choose to meet with the team several times. The team includes a D&I faculty member (AQ or JM), the program manager (MSM) and communications/marketing expert (SH), both of whom have MBAs, an implementation scientist (FR), and a research specialist (RM). These meetings focus on demand by adopters, the value proposition, and the mechanism for sustainability; how to make the innovation market ready; the budget, work plan, and timeline of various steps; in-kind support the D & I team could offer during the award period; and metrics that could be tracked for at least 3 years after the award period. The proposal requires applicants to address all eight criteria shown in Table 2. The Launchpad team also conducts independent market research with potential stakeholders to determine interest in and viability of the innovation. Launchpad staff undertake this independent assessment to obtain unbiased feedback on market demand from potential adopters. This feedback is added to the application and taken into consideration by reviewers.

## The Review Process

A multidisciplinary group of reviewers uses a process similar to that used by the NIH to evaluate grant proposals; however, the usual NIH criterion scores (Significance, Innovation, Investigators, Approach, and Environment) are replaced by the eight factors in Table 2. The reviewers are drawn from business, epidemiology, systems engineering, medical care, community organizations, the UW School of Medicine and Public Health Office of Industry Engagement, and health system administration. In the first year of the award, reviewers evaluated seven proposals; two were funded. In 2019–2020, reviewers evaluated nine proposals; three awards were made.

The review process involves two steps: (1) each reviewer receives two or three applications to assess, along with instructions, conflict of interest disclosures, and a scoring tool. (Supplementary File 2 shows the scoring tool, which is also available at www.hipxchange.org/Evidence-to-Implementation-Award). Reviewers who have a conflict with the innovation described in an application excuse themselves from reviewing that application. (2) The reviewers meet to present their reviews, discuss them, and decide on a final score for each application. For each of the eight criteria shown in Table 2, reviewers assign a score of 0 for a beginning innovation, 1 or 2 for a developing innovation, 3 or 4 for an accomplished innovation, and 5 or 6 for an exemplary innovation. Applications with the highest scores are recommended to ICTR’s leadership and the E2I funder, the Wisconsin Partnership Program, which make the final funding decision.

## Results

### E2I Awardees

The two innovations awarded E2I grants in 2018 were Tai Chi Prime (PI Betty Chewning; https://taichihealth.com/tai-chi-prime-overview/) [6] and the Wisconsin Surgical Coaching Program (PI Caprice Greenberg; https://surgicalcoaching.org/). [7–10] The 18-month award period ended for both programs on October 31, 2019. Both programs have undertaken substantial scale-up activities (described below) and now are expanding their markets through non-profit purveyors.

Tai Chi Prime aims to improve balance, mobility, and leg strength and to reduce falls among adults aged 65 years and older. The program was designed by physical and occupational therapists and Tai Chi experts. It is taught twice a week for 6 weeks, a schedule that fits well with the activity planning of most community recreational centers. Tai Chi Prime includes behavior change elements that are reinforced through the social interaction of the workshop, which is intended to build adherence to Tai Chi daily practice at home. These elements include brainstorming and reflection on doing the program at home and integrating the moves into daily life, the use of exercise logs, and streamlined content (with only eight movements).

Before Tai Chi Prime applied for the E2I award, it had been shown in a randomized, wait-list controlled trial with 206 adults aged 65 years and older to significantly improve mobility and gait, leg strength, and balance. [6] It also improved executive function and confidence about stability during activities that required balance. By the last week of class, participants practiced an average of 26 min a day.

In its E2I proposal, the principal investigator, Dr. Chewning, proposed using E2I support to develop training and implementation packages, to conduct market research and develop a marketing plan, and to develop a sustainable business plan.

The WI Surgical Coaching Program was designed to improve the skills and performance of surgeons, and therefore the quality and safety of surgical care. The program arose from evidence that most surgical errors are technical and occur in the operating room, suggesting that the most important strategy for reducing adverse outcomes may be improving the proficiency of the surgeon. The WI Surgical Coaching Program uses a peer-coaching model. It is based on principles of adult learning, addressing technical, cognitive, and interpersonal skills. It emphasizes the surgeon’s power to make changes in practice. Pilot studies had demonstrated the acceptability and perceived value of peer coaching among surgeons, and the model has been studied in two R01 grants, one complete and the other underway as of this writing. The WI Surgical Coaching Program E2I proposal aimed to prepare the way for the program to become national by conducting market research and developing a marketing campaign and promotional materials. Independent of the E2I award, the Academy of Surgical Coaching (a non-profit organization) was established as the purveyor of the program. Table 3 shows the activities conducted by each awardee during the 18 months of E2I support.

**Table 3.** Activities undertaken by Evidence-to-Implementation (E2I) awardees

Domain	Tai Chi Prime	Wisconsin Surgical Coaching Program
Development of content and infrastructure	• Leader training and class materials were finalized • Promotional materials were created • Website material was completed • A paper about Tai Chi Prime was published in a high-impact journal [6] and a presentation made to a prestigious audience at Harvard Medical School	• A partnership was formed with JCD Advisors, a coaching firm, to train coaches • Strong relationships were formed with six surgical professional societies, and interest created through presentations in making coaching part of certifying practicing surgeons • The first surgical coach training took place, as well as the first occurrence of being exhibitor at a surgical meeting • Planning had begun for 2020 Surgical Coaching Summit
Market research and development of marketing plan	• Goal set to increase trainers and course leaders regionally and nationally • Target adopters (organizations) and implementers (course leaders) identified; recruitment materials and templates created (emails, flyers, etc.) • Course leader training enrolled participants from four states • Courses were offered in six states through various venues, with more courses planned	• Market research was conducted with 63 practicing surgeons, resulting in changes to the business plan
Creation of a sustainable business plan (including purveyor, intellectual property determinations, etc.)	• The National Council of Older Adults certified Tai Chi Prime, making it eligible for funding from the Older Americans Act • A train-the-trainer program was instituted • Fidelity checks were conducted during courses, which showed high fidelity • Website material was completed • Tai Chi Health was selected as the purveyor of Tai Chi Prime	• The original plan to license the program was revised once the value of wraparound services (coach-surgeon pairing, scheduling, etc.) was recognized • The curriculum for training surgeons to be surgical coaches was certified by the UW Interprofessional Continuing Education Partnership, and efforts were begun to seek external accreditation • A comprehensive financial model was created

When PIs receive an E2I award, they must attest that they understand relevant conflict of interest rules and will comply with them. In addition, PIs must agree to submit D & I metrics yearly for 3 years after the 18-month post-award period. The first post-award metrics will be reported in 2021 for the year 2020. Metrics reported for Tai Chi Prime will include the number of leaders training, workshops held, and participants taking the workshops, both in Wisconsin and outside the state. For the Wisconsin Surgical Coaching Program – now named the Academy of Surgical Coaching – metrics will include the number of coaches trained; number of surgeons trained; and number of hospitals, health systems, surgical societies, and industry partners using the program.

Three innovations received E2I awards in the second round in 2019–2020: I-SITE: Implementation for Sustained Impact in Teleophthalmology; MOVIN: Mobilizing Older adults Via a systems-based Intervention; and Staying Healthy After Childbirth. I-SITE, led by principal investigator Yao Liu, aims to increase diabetic eye screening rates and prevent blindness by using teleophthalmology to address limited access to eye care, especially in rural areas. [11] The program guides primary care clinics through a complex implementation process to enable them to screen patients using eye cameras. MOVIN, led by principal investigators Barb King and Lindsey Steege, aims to increase opportunities for patient ambulation in hospitals by addressing barriers that prevent nurses from getting patients to walk and shifting nursing staff behavior and unit culture from mobility restriction to mobility promotion. [12] Staying Healthy After Childbirth is a telehealth/remote patient monitoring intervention for women with postpartum hypertension led by principal investigator Kara Hoppe. [13] A team of trained nurses operates as a centralized managing team to standardize and improve care.

### Program Evaluation

In May 2019, an outside evaluator collected feedback from the first two awardees about the value of the D & I services they had received. Awardees were asked about the quality of the services they had received, their confidence that their interventions would be successfully implemented and disseminated, and suggestions they had for improving the E2I grant mechanism. Both awardees were enthusiastic about the help they received from the D & I Launchpad related to business concepts and models, marketing, budgeting, and legal issues. An interview with Tai Chi Prime principal investigator Betty Chewning is here: https://youtu.be/CWfk2VsTmhs The following quotation comes from a co-investigator on the surgical coaching project:

*How does it work to run a business? We’re not business people. What groups do we need to target, helping us price our products, creating our business model, and helping us formulate that, develop that, and amend it as we’ve gone along, helping us with our pitches to [business organizations] and to other groups, our proposals to surgical societies or to health systems. We’re so used to presenting it as research, but it’s a different angle.*

Both awardees expressed confidence that their interventions will continue to be scaled up, though both recognize hurdles. Both awardees had suggestions for improving the E2I award process, such as lengthening the time between the pre-proposal and final proposal and clearly defining the roles of the D & I Launchpad team and principal investigators as proposals are developed. These suggestions were implemented during the second round of E2I awards.

## Discussion

The first two awardees of E2I awards are continuing to scale up their innovations as of this writing, three years after they received their E2I awards, a significant milestone given that approximately 95% of new ventures fail in the market. [14] Both PIs used resources available from the award to take important next steps in scaling up their innovations. Both awardees developed value propositions, marketing materials, and content for websites; engaged new sponsors and partners; were certified by important external organizations; worked out issues related to intellectual property; and identified purveyors for their innovations (an existing external organization in one case and a newly created 501(c)3 in the other). Tai Chi Prime developed training materials for various audiences, which the PI had identified in her application as critical. The Academy of Surgical Coaching focused many of its activities on marketing (e.g., developing relationships with sponsors and partners, conducting market research, creating a stronger social media presence).

### Lessons Learned

The E2I process will evolve over time, as it already has between its first and second rounds. For instance, we learned that we need to play more of a consultative role to potential grantees in the application process rather than playing an active role in application development; we evolved a much closer relationship with our University’s office of industrial relations, in recognition of the need to engage early with issues around potential conflicts of interest; and we included metrics around diversity in terms of the end users served by awardees. Nevertheless, the program’s essential elements are theoretically and empirically grounded, fill an important gap at research universities, were identified in response to what worked and did not work in a previous award program, and apply an approach to forecasting the success of entrepreneurial ventures that has demonstrated predictive validity. [5] We see the foundational elements of the program as these: giving academic researchers who have developed innovations substantial, long-term assistance with business concepts; using Critical Factor Assessment to evalute proposals, which has proven valuable in predicting the viability of commercial ventures in prior research outside health care; using a multidisciplinary team that includes experts in business and D & I science to help investigators develop their applications and move their innovations toward scale-up; using a review process comparable to the NIH review of grant proposals but geared toward an entrepreneurial mentality; engaging reviewers who have expertise in business, health care systems administration, public health, health care consulting, engineering, and other fields; and having committed principal investigators and teams who believe in the potential of their interventions, understand the requirements of scale-up, and agree to persist through the process.

Every research university has policies regarding conflicts of interest, establishing commercial enterprises (for-profit or non-profit), and working with third parties to disseminate interventions developed at the institution. These policies may also include rules regarding what type of funding was used to develop the intervention (federal, state, private, etc.). The D&I Launchpad team works closely with offices on campus regarding potential conflicts of interest. We have processes in place, like mandatory investigator consultations with the Office of Industry Engagement, to review work plans. To initiate similar programs at other CTSAs, close collaboration with conflict-of-interest entities is critical.

### Limitations

A program like the E2I award requires substantial funding. At UW, ICTR leaders recognized the need to focus effort on helping investigators scale up their non-patentable innovations. They channeled investment into the infrastructure required to support a robust D&I presence, including earmarking funds for the E2I award from the Wisconsin Partnership Program, an entity established in 2004 through an endowment gift from Blue Cross and Blue Shield United of Wisconsin’s conversion to a stock insurance corporation. To replicate a program like the E2I program, CTSA leaders will need to: (1) Prioritize the need to support D&I activities like the E2I program and (2) Be resourceful in procuring funds to support the program. Finally, the E2I program is an infrastructure development stage, with the first two applications funded by the program just reaching maturity at manuscript submission. Though empirically based, it is too early to tell if the Critical Factors Assessment approach adapted for the E2I program can accurately predict the future success or failure of programs funded through the award.

### Future Research

We are currently conducting an environmental scan of CTSAs nationwide to identify programs similar to the E2I award. Collaboration among CTSAs offering similar programs could help maximize the impact of research supported by CTSAs by building inertia around late-stage translation and an evidence base around factors that ease or slow scale-up. Awardees are required to provide program outcomes for a period of three years. Specific metrics are customized to each program and are loosely based on the Reach, Effectiveness, Adoption, Implementation, Maintenance framework. [15] Impact metrics pertain primarily to the number of patients or consumers “reached” by each award. The requirement that awardees provide metrics about their progress for three years beyond award termination will eventually yield important information about the success of the E2I program and improvements that could be made to it, findings that could help other CTSAs strengthen similar programs at their institutions.

## Supplementary material

For supplementary material accompanying this paper visit http://dx.doi.org/10.1017/cts.2021.828. click here to view supplementary material
