# Supplementary material for: Encouraging the scale-up of proven interventions: Infrastructure development for the “Evidence-to-Implementation” award
Source: J Clin Transl Sci. 2021 Jul 26;5(1):e160. doi: 10.1017/cts.2021.828 (PMC8427544; doi:10.1017/cts.2021.828)
Supplement: Supplementary file 1 [file ctssup.zip › S2059866121008281sup001.docx]

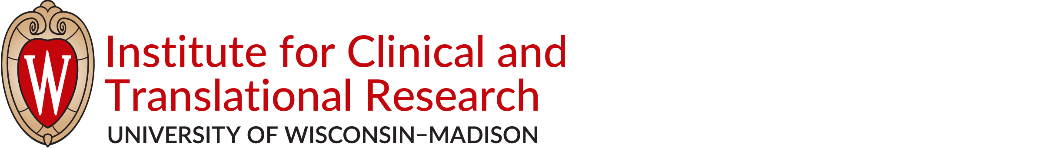


**2021 Request for Applications for the Evidence-to-Implementation Award (RFA)**

**The purpose of this award is to speed the widespread use of evidence-based health innovations developed by UW researchers.** The award is intended for investigators who have developed a successful innovation (e.g., a care model, behavioral or health intervention, digital innovation, etc.) that meets a specific demand in health care or the community and has the potential for significant impact. In addition, investigators must be:

- Committed to disseminating and implementing their work broadly
- Compliant with all UW and State of Wisconsin rules relating to conflicts-of-interest
- Willing work with the Dissemination and Implementation (D&I) Launchpad team in developing materials.
- Open to having their implementation materials disseminated and marketed publicly, such as by posting them on the HIPxChange (<https://www.hipxchange.org/)>
- Willing to have their materials showcased in D & I Launchpad materials
- Willing to work with stakeholders, partners, or purveyors to ensure that the innovation is sustained
- Willing to provide impact metrics for at least three years after the award is completed

The UW Institute for Clinical and Translational Research (ICTR) is committed to supporting the translation of research into practice. Specifically, ICTR aims to develop targeted implementation support for high-demand research through the D & I Launchpad. The D & I Launchpad is a consulting service that provides resources to increase the use of evidence-based practices. Service areas include education and training, research consultation, communication and translation packaging, and implementation support. For more information, please visit <https://ictr.wisc.edu/dissemination-implementation/>.

The Evidence-to-Implementation (E2I) Award supports ICTR’s aim by expediting the transfer or commercialization of evidence-based practices, interventions, and innovations to appropriate end users. The award supports the development of a launch package consisting of materials that support the dissemination and implementation of an evidence-based innovation or intervention. The package will be unique to each project, but may include a business plan; value proposition; marketing, sales, and financial plans; a review of the intellectual property; and memorandums of understanding (MOUs).

**E2I award specifics**

The E2I award includes up to $75,000 in direct costs plus substantial in-kind D & I Launchpad support. The award period will be determined as the launch package is developed, but will not exceed 18 months. No-cost extensions will not be considered.

- Developing a successful launch package requires the input of researchers. Thus, the award is intended support the principal investigator (PI)’s participation in this iterative process.
- The D & I Launchpad team will work closely with the applicant through the award period. In-kind support may include developing training materials and branding and marketing materials and toolkits. The goal is to disseminate the innovation at the end of the award period. The University may disseminate the innovation itself or determine that this could be more effectively accomplished by an external party. Either way, dissemination would take place in accord with University policies and practices.
- Expectations for the participation of both the applicant and the ICTR-CAP D & I Launchpad team will be specified in a mutually agreed upon work plan with milestones. Funding and in-kind support will be predicated on achieving the milestones.
- The budget proposal will be developed in consultation with the Launchpad team, and in-kind support from the Launchpad team will be detailed in the budget.
- A small test and evaluation of the launch package developed through the award will be included in the award.
- Matching funds and/or in-kind support from the applicant’s department is a positive contribution.

**Applicant eligibility**

- The PI must have faculty or scientist status at UW-Madison or Marshfield Clinic Research Institute (either sole or joint appointment).
- The PI for this award is not required to be the individual who conducted the research to establish an evidence base, although the researcher contributing to the evidence should be a collaborator in the dissemination efforts.

| **Important Application Dates** | |
| --- | --- |
| Required 60-minute application workshop   - Attend in person - Watch live stream - Watch recording | November 12, 2021 – 4:00 PM  November 13, 2021 – 12:00 PM  The workshop will be livestreamed and recorded.  Livestream: live.video.med.wisc.edu  Recording: videos.med.wisc.edu |
| Pre-proposal due | December 11, 2021 |
| Step 3 applicants notified | December 18, 2021 |
| D & I team meetings with applicant to support the development of the application. One consultation is required. | December 2021 – January 2022 |
| First draft of application due | January 14, 2022 |
| Final application due | February 1, 2022 |
| Review, scoring, and in-person review meeting by D & I council completed | February 15, 2022 |
| Award announced | February 22, 2022 |
| Award start date | March 1, 2022 |

**Application Process and Components**

**Step 1: Workshop**

Attend the workshop to learn about the E2I award. The workshop will describe the E2I award processes, application, and award timeline. The workshop will also describe the required components of the pre-proposal and proposal and the restrictions on using E2I and University funds. If you are unable to attend the workshop in person, you may watch it via live streaming or a post-workshop recording. Applicants are required to attend the workshop.

**Step 2: Pre-proposal**

The pre-proposal should be no longer than 3 pages, in 11-point Arial with 1-inch margins. At the top, include your name, departmental affiliation, position title, and contact information. The pre-proposal and proposal should be written clearly and concisely for a lay audience, with minimal jargon and technical language. The pre-proposal and proposal address the same criteria, shown below.

| **Criteria for E2I pre-proposals and proposals and for review** |
| --- |
| **1. Features & benefits**   - Does the innovation offer performance advantages compared with current solutions? - Would the innovation create value for implementing organizations? - Is the innovation evidence-based or does it have scientific merit? |
| **2. Market readiness**   - Has the innovation gone through different developmental stages? - What steps have been taken to prepare for broad dissemination? |
| **3. Competition and barrier to entry**   - What will create an ongoing competitive advantage for the innovation? - What is unique about it? |
| **4. Demand/adoption/target customer**   - Has an adopter or first customer been identified? - Have potential adopters or customers been involved in development? - Is there evidence of demand? |
| **5. Sustainability – purveyors and partners**   - Is a purveyor willing to represent the product and take it to new customers? - Are partners willing to collaborate with the investigator? |
| **6. Potential for impact**   - Will the innovation impact the health of individuals, improve care processes, and/or increase safety or efficiency? - Is the innovation likely to benefit a large audience or a subset of the population? |
| **7. Investigator and team**   - Do the investigator and team have the expertise, resources, ability, and motivation to navigate the challenges to optimize uptake of the innovation, as evidenced by previous experience? |
| **8. Sustainability – finances**   - Is the innovation feasible? Is it acceptable to target audiences? - Does it have the potential to sustainably produce revenue for the purveyor? |

The pre-proposal should be an abstract of your project. Try to address each of the eight criteria. Please number each response. We understand that you may not be able to address all the factors in the pre-proposal, or address them completely, but trying to answer them will show both you and the D & I Launchpad staff who review the pre-proposal criteria what additional work may be required to complete the proposal and during the award period. In criteria 5, *purveyor* means an organization that spreads an innovation through marketing and training others to adopt and implement the innovation. A purveyor provides technical assistance to maximize fidelity.

Submit your pre-proposal as a single PDF or Word document via email to [di-launchpad@ictr.wisc.edu](mailto:di-launchpad@ictr.wisc.edu) no later than December 11, 2021. Please indicate whether you attended the workshop in person or watched it via live streaming or as a recording.

**Step 3: Proposal**

Applicants whose pre-proposals meet the goals of the E2I program will be invited to develop proposals. This step includes at least one meeting with the D & I Launchpad team to consult about the proposal and discuss how the team might help the applicant both in developing the proposal and during the award period, if the proposal receives an award. We recommend that applicants have more than one meeting. The meeting or meetings have three goals: (1) to discuss responses to the eight criteria, focusing on the demand by adopters, a value proposition, how the innovation can be sustained, and anything that needs to be done to ensure that the innovation will be ready for dissemination; (2) the in-kind support the Launchpad team can provide; and (3) key metrics about the impact of the innovation that can be tracked for at least three years after the award period.

The final proposal must be submitted as a single PDF or Word document via email to [di-launchpad@ictr.wisc.edu](mailto:di-launchpad@ictr.wisc.edu) no later than February 1, 2021.

**Review Process**

**Pre-proposal review**

The D & I Launchpad Manager will review pre-proposals for completeness and send screened pre-proposals to the D & I Launchpad team. The Launchpad team will then review pre-proposals for their alignment with the goals of the program and invite investigators whose pre-proposals align well to submit proposals.

**Proposal review**

The UW ICTR-CAP D & I Council will review proposals and recommend funding to ICTR leaders. Council members consist of the UW-Madison faculty and staff and community members with expertise in health care, public health, and business. Council members will begin by reviewing proposals and generating a list of questions for each applicant. The D & I Launchpad team will consolidate questions from reviewers and email them to applicants, who will have 48 hours to respond. The Council’s final recommendations will be decided upon during an in-person review meeting. All applicants will receive de-identified reviewer comments.
