# Supplementary material for: Encouraging the scale-up of proven interventions: Infrastructure development for the “Evidence-to-Implementation” award
Source: J Clin Transl Sci. 2021 Jul 26;5(1):e160. doi: 10.1017/cts.2021.828 (PMC8427544; doi:10.1017/cts.2021.828)
Supplement: Supplementary file 1 [file ctssup.zip › S2059866121008281sup002.docx]

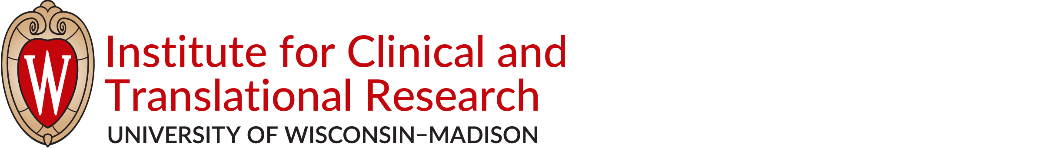


**2021 Evidence-to-Implementation Reviewer Instructions and Scoresheet**

This scoresheet provides:

- eight criteria from the Critical Factors Analysis, an evidence-based set of criteria for predicting the success of new ventures developed by the Canadian Innovation Centre.^1^ Please assess the product or service against each of these criteria, using the guiding statements to make the assessment.
- a place to enter an overall rating
- a comment section for listing the innovation’s most important strengths and weaknesses.

| **Criteria** | **Beginning**  **0** | **Developing**  **1-2** | **Accomplished**  **3-4** | **Exemplary**  **5-6** | **Score** |
| --- | --- | --- | --- | --- | --- |
| **1. Features & benefits**   - Does the innovation offer performance advantages compared with current solutions? - Would the innovation create value for implementing organizations? - Is the innovation evidence-based or does it have scientific merit? | The product or service does not have advantages over current practice and scientific justification is lacking. | The product or service matches the performance of currently deployed solutions at a competitive cost. | The product or service has measurable benefits over current solutions or offers substantive advantages in a market niche.  The science behind the innovation is sound and the product has been developed and tested with, at minimum, excellent pilot data. | The product or service has substantial advantages over current solutions, or there is no comparable solution in existence.  The science behind the innovation is sound. The innovation is finished, and the product has been satisfactorily tested. |  |

| **2. Market readiness**   - Has the innovation gone through different developmental stages? - What steps have been taken to prepare for broad dissemination? | The product or service is not well described. | The concept of the innovation has been established, but it still needs to be developed and tested. | The innovation has gone through different stages of product development. Dissemination and implementation packages still need to be tested for effectiveness. | The innovation or product or service is finished, it has been tested, the implementation package has been shown to be effective, and a dissemination plan has been developed. |  |
| --- | --- | --- | --- | --- | --- |
| **3. Competition and barrier to entry**   - What will create an ongoing competitive advantage for the innovation? - What is unique about it? | The product or service has significant existing competition that delivers equal or superior results. | The product or service is innovative, but there are limited formal mechanisms to reduce the likelihood that competitors will replicate key features in the future. | The product or service embeds unique knowledge or know-how that might be patentable, or has another unique property that makes it challenging but not impossible for others to replicate. | The product or service has received a patent or embeds proprietary technology or knowledge that is not easily replicated. Alternatively, the innovation has a unique feature (such as a brand) that creates a significant barrier to competitors. |  |

| **4. Demand –adoption – target customer**   - Has an adopter or first customer been identified? - Have potential adopters or customers been involved in development? - Is there evidence of demand? | At present, the product has not been independently validated, no adopter or first customers have agreed to test or purchase it, and/or it does not meet an important need based on reviewer experience. | Meets an important need based on reviewer understanding of the healthcare system. | Market research has confirmed a specific market for the product or service, but no commitments have been made to date to test or purchase the product. | Customers have been intimately involved in the development process and have committed either to purchase or try the product or service as soon as it becomes available. |  |
| --- | --- | --- | --- | --- | --- |
| **5. Sustainability –purveyors and partners**   - Is a purveyor willing to represent the product and take it to new customers? - Are partners willing to collaborate with the investigator? | No dissemination and implementation purveyors or partners have been identified. | At this point, although distribution or dissemination partners have been identified, they have not been approached. | Possible distribution and dissemination purveyors and partners have been identified and initial discussions held. Potential purveyors and partners are interested, but no formal agreements are in place with these partners. | Critical distribution or dissemination purveyors or partners have been engaged in the development process and have made firm commitments to participate as soon as the product or service is ready to scale up. |  |

| **6. Potential for impact**   - Will the innovation impact the health of individuals, improve care processes, and/or increased safety or efficiency? - Is the innovation likely to benefit a large audience or a subset of the population? | The innovation is estimated to have a low impact and is very unlikely to reach the target audience. | The innovation is estimated to have a modest impact and reach a modest proportion of the target audience. | The innovation is estimated to have a moderate impact and reach a moderate proportion of the target audience. | The innovation has the potential to have a great impact on individuals or health system processes and a high enough proportion of the target audience can be reached to achieve this impact. |  |
| --- | --- | --- | --- | --- | --- |
| **7. Investigator and team**  Do the investigator and team have the expertise, resources, ability, and motivation to navigate the challenges to optimize uptake of the innovation, as evidenced by previous experience? | Experience and commitment to implementation is not evident. | The PI and/or team members’ experience is primarily technical, or limited, and provides no direct evidence that it will help them support the implementation and dissemination of their innovation. The PI lacks commitment and/or the capacity to assist with dissemination, including a handoff to a purveyor. | The PI and/or team members’ have significant skills and experience that are not directly relevant but will help support their success. The PI is only moderately committed to seeing the process through to dissemination and handoff to a purveyor, or the PI or team has some capacity challenges. | The PI and/or team members have deep and significant relevant experience that will support their success and are very committed to seeing the process through to dissemination, including handing off the innovation to a purveyor, and have the capacity to assist with dissemination. |  |

| **8. Sustainability – finances**   - Is the innovation feasible? Is it acceptable to target audiences? - Does it have the potential to sustainably produce revenue for the purveyor? | No business model exists or could probably be developed to sustainably spread the innovation. | A business model may be able to be developed that shows how the spread of the innovation could be sustainably paid for. | A business model could reasonably be developed that shows how the spread of the innovation could be sustainably paid for. | A business model can undoubtedly be developed that shows how the spread of the innovation could be sustainably paid for. |  |
| --- | --- | --- | --- | --- | --- |

^1^ [**https://innovationcentre.ca/services/services-for-entrepreneurs/critical-factors-assessment/**](https://innovationcentre.ca/services/services-for-entrepreneurs/critical-factors-assessment/) See also Astebro T, Elhedhli S. The effectiveness of simple decision heuristics: forecasting commercial success for early-stage ventures. *Management Science* 2006; 52: 395-409.

**9. Overall rating.** Please use the same 0-6 scale as used for the eight criteria.

**10. Comments.** Please provide a bulleted list of strengths and weaknesses.

Strengths:

Weaknesses:
